# In situ gold adsorption experiment at an acidic hot spring using a blue-green algal sheet

**DOI:** 10.1038/s41598-024-56263-3

**Published:** 2024-03-08

**Authors:** Tatsuo Nozaki, Yasuyuki Fukushima, Satoshi Okada, Yutaro Takaya, Akiko Makabe, Masayuki Watanabe

**Affiliations:** 1 Research Institute for Marine Resources Utilization, Submarine Resources Research Center, Japan Agency for Marine-Earth Science and Technology (JAMSTEC), 2-15 Natsushima-cho, Yokosuka, 237-0061 Japan; 2 School of Engineering, Frontier Research Center for Energy and Resources, The University of Tokyo, 7-3-1 Hongo, Bunkyo-ku, Tokyo, 113-8656 Japan; 3 Department of Planetology, Graduate School of Science, Kobe University, 1-1 Rokkodai-cho, Nada-ku, Kobe, 657-8501 Japan; 4 Department of Earth and Environmental Sciences, Faculty of Science, University of Ottawa, 25 Templeton Street, Ottawa, ON K1N 6N5 Canada; 5 Applied Physics and Chemistry Group, Technology Platform Center, Technology and Intelligence Integration, IHI Corporation, 1 Shin-nakahara-cho, Yokohama, 235-8501 Japan; 6 Super-cutting-edge Grand and Advanced Research Program, Japan Agency for Marine-Earth Science and Technology (JAMSTEC), Institute for Extra-cutting-edge Science and Technology Avant-garde Research (X-Star), 2-15 Natsushima-cho, Yokosuka, 237-0061 Japan; 7 Department of Systems Innovation, School of Engineering, The University of Tokyo, 7-3-1 Hongo, Bunkyo-ku, Tokyo, 113-8656 Japan; 8 Faculty of Science and Engineering, Waseda University, 3-4-1 Okubo, Shinjuku-ku, Tokyo, 169-8555 Japan

**Keywords:** Water microbiology, Geochemistry, Mineralogy

## Abstract

Gold (Au), as one of the most precious metal resources that is used for both industrial products and private ornaments, is a global investment target, and mining companies are making huge investments to discover new Au deposits. Here, we report in situ Au adsorption in an acidic hot spring by a unique adsorption sheet made from blue-green algae with a high preferential adsorption ability for Au. The results of in situ Au adsorption experiments conducted for various reaction times ranging from 0.2 h to 7 months showed that a maximum Au concentration of 30 ppm was adsorbed onto the blue-green algal sheet after a reaction time of 7 months. The Au concentration in the hot spring water was below the detection limit (< 1 ppt); therefore, Au was enriched by preferential adsorption onto the blue-green algal sheet by a factor of more than ~ 3 × 10^7^. Thus, our gold recovery method has a high potential to recover Au even from an Au-poor solution such as hot spring water or mine wastewater with a low impact on the environment.

## Introduction

The contemporary world gold reserves are estimated to be about 52000 tons^1^, which corresponds a volume of less than that of one Olympic-sized swimming pool. At present, gold is produced mainly from epithermal Au–Ag, placer, and orogenic gold deposits, as a biproduct from porphyry copper (Cu), volcanogenic massive sulfide, or other hydrothermal deposits^2–6^, and by recycling from the urban mine. The total annual Au production from these sources was 3090 tons in 2021 and 3100 tons in 2022^1^; thus, assuming the same reserve and annual production rate in the future, the reserve to production ratio can be estimated to be only about 17 years. For this reason, mining and metal companies are investing large amounts of money to discover new Au deposits to ensure a stable and diversified supply of Au resources. As the number of newly discovered large metal deposits has decreased, biosorption techniques for metal elements such as Cd, Cu, Ni, Pb, and Zn have become attractive for recovering metal elements with a low impact on the environment^7–11^. In particular, some blue-green algae with a high preferential adsorption ability for Au have been utilised in biosynthesis experiments to produce gold nanoparticles^12–15^.

**Figure 1:**
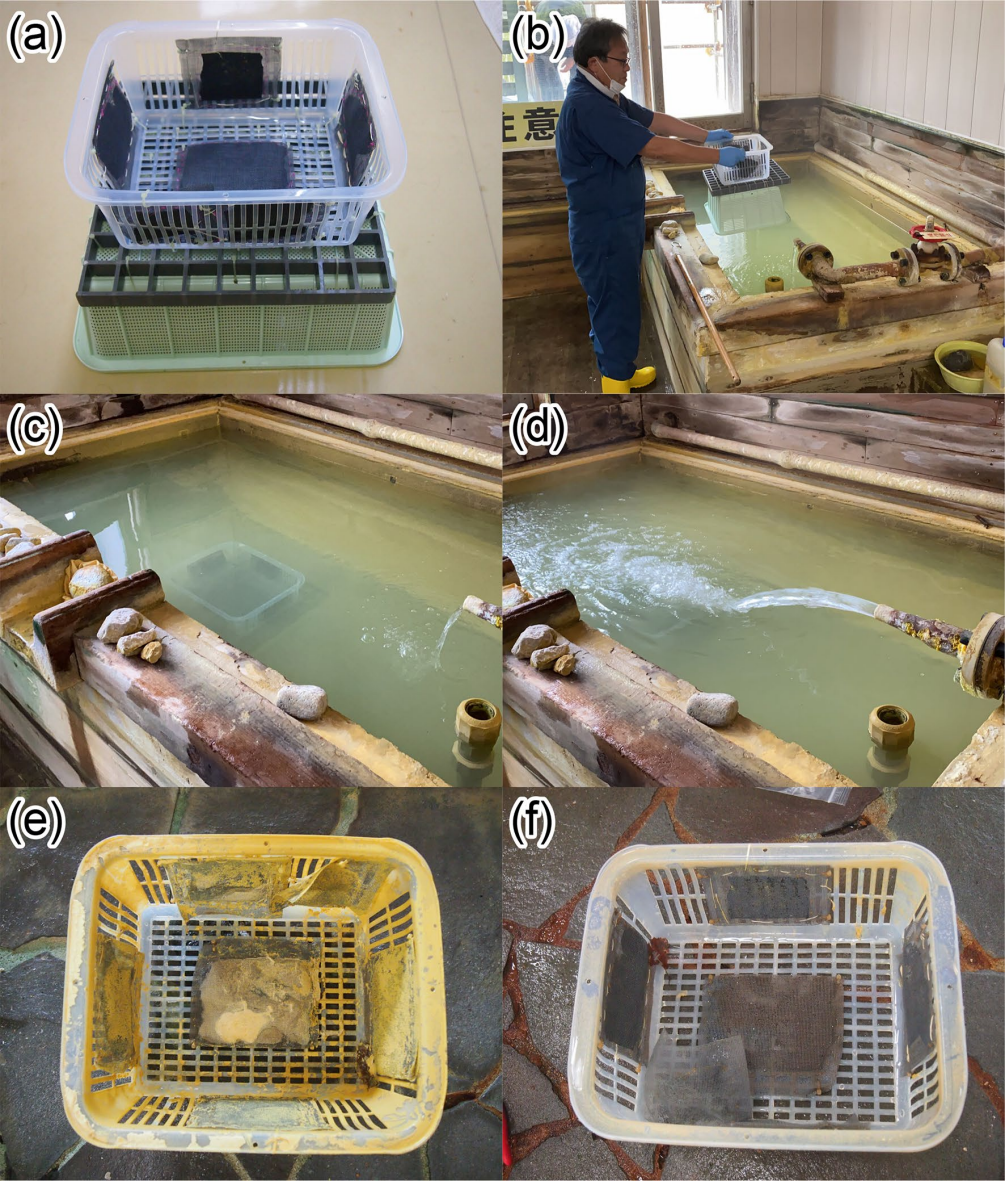
In situ Au adsorption experiment. (**a**) Blue-green algal sheets were attached to the bottom and sides of a PFA basket (30 cm by 25 cm by 15 cm high). Then, the PFA basket was set on an overturned plastic basket to prevent the blue-green algal sheets from being buried under hot spring water precipitate (sinter deposits). To prevent corrosion and contamination, no metal pieces were used. (**b**–**d**) Photographs taken (**b**) just before and (**c**, **d**) after the blue-green algal sheets and PFA basket were put into the test water tank at the Neutralisation Treatment Facility at Tamagawa Hot Spring. Hot spring water with a temperature of 60 °C and a pH of 1.2 flowed through the tank at a rate of 6 m^3^/h. (**e**) The blue-green algal sheets after the 7-month in situ Au adsorption experiment in the test water tank. The yellowish precipitate is a sinter deposit dominated by native sulfur. (**f**) The blue-green algal sheets just after they were washed with tap water to remove the sinter deposit.

Filamentous blue-green algae living in alkaline and Na-Si-Ca-S-K-B-rich hot spring waters in northeastern Japan with temperatures ranging from 50 to 60 ℃ and a pH of 8–9 are known to have a high preferential adsorption ability for Au^16–18^. These blue-green algae, which have been classified into the genus *Leptolyngbya* by 16S ribosomal DNA analyses and BLASTn (Nucleotide Basic Local Alignment Search Tool) searches, are possibly *L. laminosa*^18^. The maximum amount of Au adsorption has been estimated to be 0.31 kg per kg algae, and Au was well adsorbed even onto dead, desiccated algae^16–18^. We thus hypothesised that a sheet of dry, dead algae could be used as a concentrator for Au, even from an Au-poor solution. We therefore conducted in situ experiments in which we used algal sheets for Au adsorption (recovery) from acidic hot spring water (Fig. 1a), such a trial result about the Au recovery from acidic hot spring water has been quite limited so far. At the end of the longest in situ adsorption experiment of 7 months, the Au concentration on the blue-green algal sheet was up to about 30 ppm; this concentration is much higher than that of the typical cut-off grade (6–8 ppm^19^) for Au deposits on land.

### Tamagawa hot spring

The in situ Au adsorption experiments were performed in a test water tank located in the Tamagawa Neutralisation Treatment Facility, Tamagawa Hot Spring, Akita Prefecture, northeastern Japan (Fig. 1b–d). Tamagawa Hot Spring has been active for more than 300 years. It is one of the most acidic hot springs in Japan, with a temperature of 98 ℃, a pH of 1.2, a discharge rate of 9000 L/min, and a heat discharge of 1.8 × 10^15^ J/year^20–24^. The hot spring is associated with an old sulfur mine^25^ and with occurrences of the rare mineral hokutolite [(Ba,Pb)SO_4_]^26^. It is rich in both Cl and SO_4_, but Cl is usually dominant (for example, at Obuki, the largest discharge site, Cl = 4020 ppm and SO_4_ = 1730 ppm in 2007^23^). From its oxygen (δ^18^O) and hydrogen (δD) isotopic compositions, the hot spring water at Obuki has been inferred to experience boiling (phase separation) at several tens to several hundreds of meters below ground^20,23^. It also contains volcanic gas derived from deeper magma with an estimated mixing ratio of volcanic gas to meteoric water of 1:5^20,23^. We chose Tamagawa Hot Spring as the site for our in situ Au adsorption experiment because the solubility of gold is known to increase exponentially with increasing temperature and decreasing pH^27,28^.

The acidic hot spring water is neutralised by lime (pH > 3) at the Neutralisation Treatment Facility to protect the flora and fauna in Tamagawa Dam Lake and downstream^24^. Because it was not possible to conduct in situ Au adsorption experiments at the Obuki hot spring source, we used a test water tank at the Neutralisation Treatment Facility, which is about 600 m downstream from the hot spring source (Fig. 1b–d). The temperature and pH of the hot spring water flowing into the test water tank are 60 ℃ and 1.2, respectively^29^.

## Materials and methods

To make algal adsorption sheets for Au, incubated blue-green algae were washed with tap water, a hydrochloric acid solution, and then tap water again, followed by oven-drying^17,18^. The blue-green algae for the adsorption sheets were packed into a fiberglass mesh, and the adsorptions sheets were then attached to the bottom and sides of an acid-resistant, perfluoroalkoxy (PFA) basket by nylon strings without any metal parts, which are easily corroded and destroyed by the acidic hot spring water. The PFA basket was placed on an overturned plastic basket to prevent burial of the Au adsorption sheets by hot spring water precipitates (sinter deposits). The basket with the Au adsorption sheets was immersed in a test water tank through which hot spring water flowed at a rate of 6 m^3^/h and the water above the certain level flowed out from the tank via the standpipe (Fig. 1d) for various treatment periods (0, 0.2, 0.5, 1, 3, 6, and 13 h; 1, 2, 3, 4, 5, 6, and 7 days; 2 and 3 weeks; and 1, 2, 3, and 7 months) so that secular changes in the adsorbed element concentrations could be observed. After each in situ experiment, the sheets were immediately washed with tap water to remove the yellowish, sulfur-rich precipitate from the surface (Fig. 1e, f).

The algal adsorption sheets were then brought back to the laboratory, where they were washed well again with tap water, dried, and then split into two parts, together with the removal of the fiberglass mesh. One part was used for microscopic observations, and the other was pulverised for geochemical analysis. The surface structure, texture, and precipitated minerals on the adsorption sheets were observed by field emission-scanning electron microscopy (FE-SEM) and energy-dispersive X-ray spectroscopy (EDS)^30^. The element concentrations on the adsorption sheets and in the precipitate were determined by inductively coupled plasma-mass spectrometry (ICP-MS) with the mixed acid digestion method^31,32^ (Supplementary Table S1). The chemical composition of the hot spring water source at Obuki was determined by ion chromatography (IC) and ICP-atomic emission spectrometry (ICP-AES)^33^ (Supplementary Table S2), and the chemical composition of the hot spring water in the test water tank was determined by ICP-MS at Japan Agency for Marine-Earth Science and Technology (JAMSTEC)^34^ (Supplementary Table S3) and by ICP-MS and high-resolution (HR)-ICP-MS at Activation Laboratories Ltd., a commercial analytical service (Supplementary Table S4).

## Results and discussion

### SEM observations of the adsorption sheets

**Figure 2:**
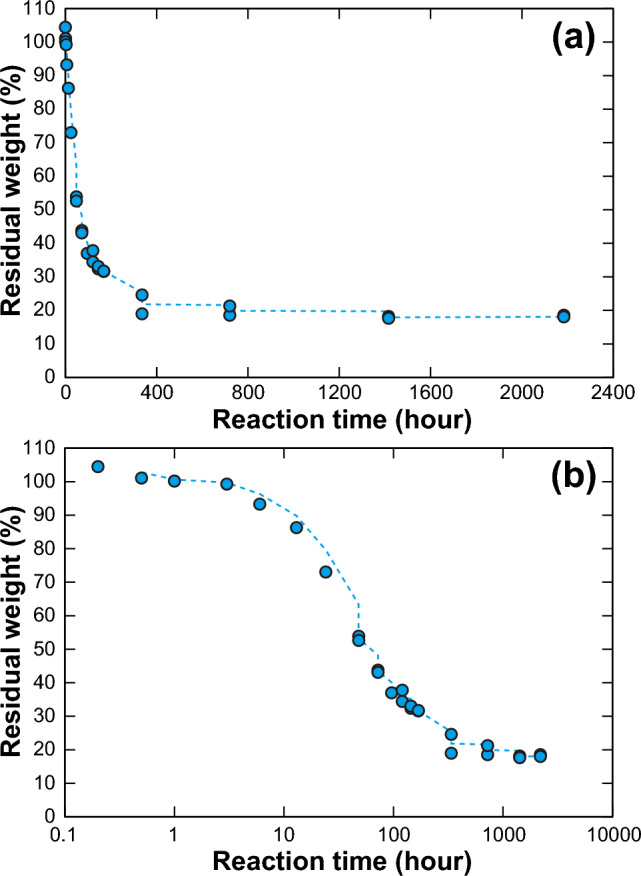
Residual weight (relative to the starting weight) of the blue-green algal sheets in relation to reaction time. In (**a**), the horizontal axis scale is linear, and in (**b**), it is logarithmic. The blue dashed lines represent the two-point moving average.

During the in situ Au adsorption experiment, the blue-green algal adsorption sheets became smaller and thinner than the starting material, and their dry weights decreased exponentially with the reaction time (Fig. 2). At 24 h, the weight of the adsorption sheets had decreased to 73% of that of the starting material (a 27% weight loss), and at 168 h (1 week), 68% of the weight had been lost (Fig. 2). At 2 weeks, the weight loss had slowed and almost plateaued, and at 2–3 months, only 18–19% of the original weight remained. No weight change data are available from the 7-month experiment (Fig. 2). In addition to the weight loss, the surface structure and texture of the adsorption sheets changed with time (Supplementary Figs. S1 and S2). Initially, clear filamentous and tube-like structures ~ 1 μm in diameter derived from the blue-green algae could be observed by FE-SEM, but after one week, these structures became vague and unclear (Supplementary Figs. S1 and S2a, b). After 7 months, the filamentous structures became fragmented, and the fragments then aggregated and were partly covered by the precipitated minerals; the end result was a massive sheet-like structure (Supplementary Figs. S1 and S2c, d).

**Figure 3:**
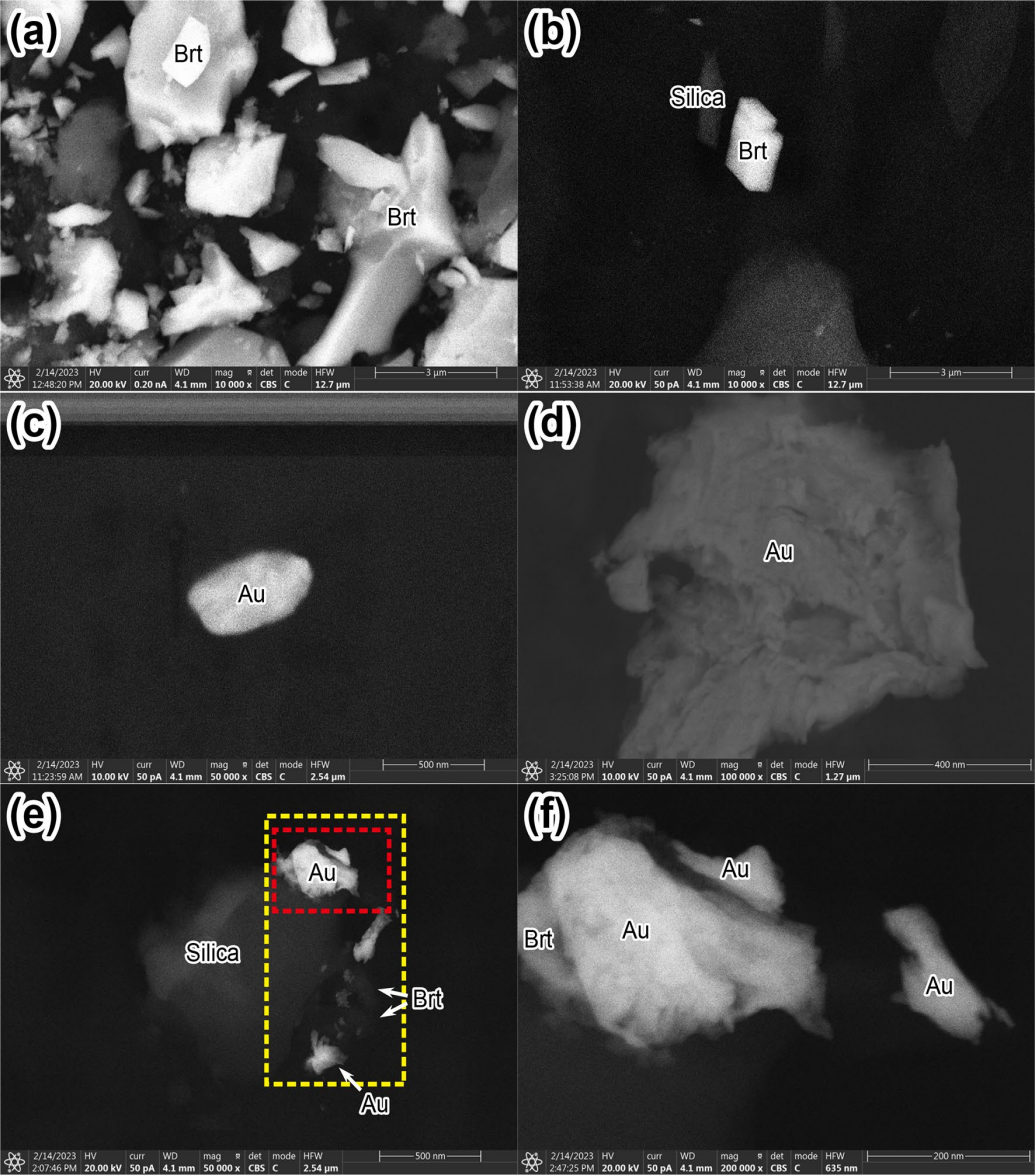
SEM images of a blue-green algal sheet after the 7-month in situ Au adsorption experiment. (**a**) Abundant barite grains. (**b**) A euhedral barite grain with a silica mineral grain. (**c**–**f**) Au-rich grains formed on the blue-green algal sheet. (**f**) An enlarged image of the grain within the red dotted rectangle in (**e**). The yellow dotted square indicates the area of the SEM–EDS elemental mapping shown in Fig. 4c. Brt, barite; Au, Au-rich grain.

**Figure 4:**
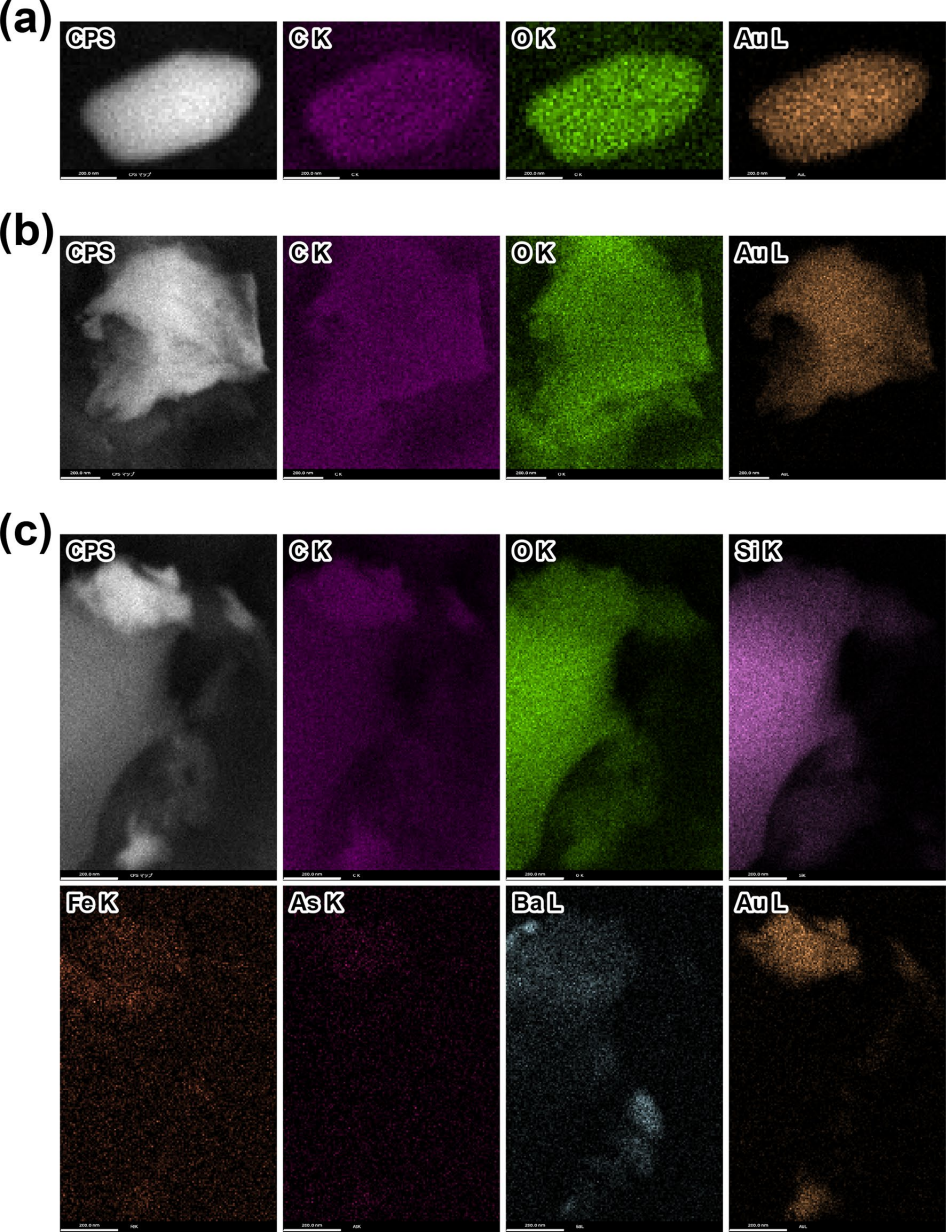
SEM–EDS elemental mappings. (**a**–**c**) Mapping results for the Au-rich grains shown in Fig. 3c–e. The EDS elemental maps were obtained from the L lines for Ba and Au and from the K lines for other elements. X-ray counts per second (CPS) maps were also obtained. White scale bars indicate 200 nm.

The major constituent minerals observed on the blue-green algae sheet were determined by SEM–EDS to be barite (BaSO_4_), halite (NaCl), and a silica mineral, along with minor unidentified Ca and Fe silicates, Fe-oxide (or oxyhydroxide?), As-S minerals, and galena (PbS) (Fig. 3a, b and Supplementary Fig. S3). Among these minerals, barite was the most abundant and exhibited a euhedral shape and high crystallinity (Fig. 3a, b). The silica mineral species could not be determined only by SEM–EDS observation, but in hot spring water with a pH of 1.2 and a temperature of 60 ℃, opal plausibly precipitates, as at Shinyu, Tateyama Hot Spring, Toyama Prefecture, Japan^35^. The Au-rich grains were not euhedral but rounded (Figs. 3c and 4a) or irregular in shape, with a maximum size of ~ 1 μm (Figs. 3d–f and 4b, c). In a laboratory experiment using blue-green algae and a HAuCl_4_ solution, nano-scale Au grains with a typical size of several tens of nanometers were initially precipitated^17,18^; therefore, the presence of large Au grains of several hundred nanometers on the blue-green algal sheet indicates overgrowth of Au nano-particles (Figs. 3c–f and 4a–c). One Au-rich grain had an angular rounded shape under the FE-SEM observation (Figs. 3c and 4a). Native gold has an isotropic structure (Fm3m^36^), but this Au-rich grain does not have an isotropic morphology. The largest Au grain in our FE-SEM observations (Figs. 3d and 4b) had a rod-like, filamentous shape reflecting the continuous overgrowth of the Au grain along the original filamentous shape of the blue-green algae during the 7-month in situ adsorption experiment.

Elemental mapping by SEM–EDS (Fig. 4 and Supplementary Fig. S3) showed that the Au-rich grains did not contain any Ag, but they often included some Ni or As and S, as well as C, N, O, Al, and Si, which were partly derived from the blue-green algae themselves or due to background noise from reflected electrons. Some of the Au grains were overgrowth on barite or silica mineral grains (Figs. 3e, f and 4c). Au nano-particles with a width of 15–30 nm attached to the surface of one massive, irregularly shaped Au-rich grain (Fig. 3f) resulted from (1) the simultaneous formation of Au nano-particles (nuclei of larger Au grains) at the adsorption sheet surface or (2) overgrowth of existing Au nano-particles/grains during continuous mineralisation in hot spring water.

### Au adsorption on the blue-green algal sheets

The Cl and SO_4_ concentrations in the hot spring water source at Obuki were 94.91 mM (3365 ppm) and 14.04 mM (1348 ppm), respectively (Supplementary Table S2); thus, the SO_4_ [ppm] / Cl [ppm] ratio was ~ 0.4. The Tamagawa hot spring water is a Cl-SO_4_-mixed type, but, consistent with previous studies^21,23^, it is dominated by Cl rather than by SO_4_. The Au concentrations of the hot spring water in the test water tank determined by ICP-MS were all below the detection limit, and those determined by HR-ICP-MS at Activation Laboratories Ltd. were also below the detection limit (< 1 ppt), except for one sample (THS-UF3; Supplementary Tables S3 and S4). The concentrations of elements in the hot spring water did not differ greatly between the filtered and unfiltered samples.

**Figure 5:**
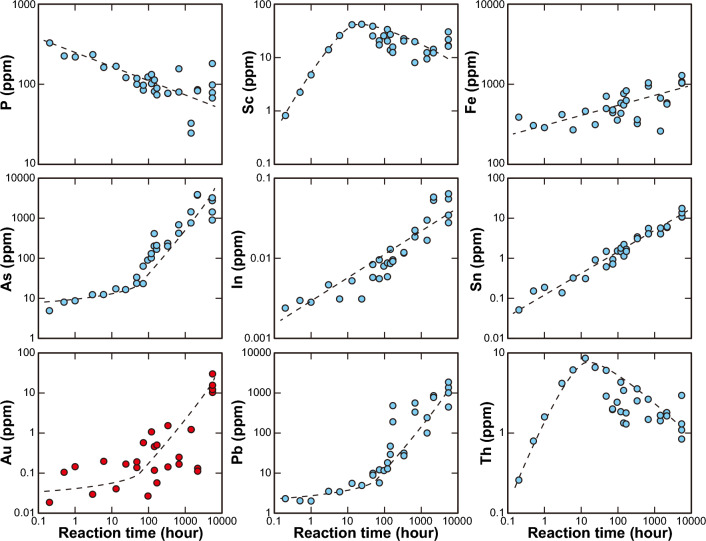
Secular changes in element concentrations. The changes with time of the concentrations of each element adsorbed onto the blue-green algal sheet show four patterns: (1) an exponential increase (As, Au, and Pb), (2) a linear increase (Fe, In, and Sn), (3) a linear decrease (P), and (4) an exponential increase followed by a gradual decrease (Sc and Th).

Bulk geochemical compositions of the blue-green algal sheets after the in situ Au adsorption experiment are shown in Fig. 5 and Supplementary Table S1. Elements with maximum concentrations exceeding 0.1 wt% (1000 ppm) after 7 months were Al (0.17 wt%), Ti (0.16 wt%), Fe (0.13 wt%), As (0.39 wt%), Ba (0.78 wt%), and Pb (0.19 wt%). The Ba concentrations in the hot spring water in the test water tank were only 0.3–0.5 ppm, much lower than those of the other major elements such as Na, Mg, Al, K, Ca, and Fe (> 20 ppm) (Supplementary Tables S3 and S4). However, on the adsorption sheets, barite was the most abundant mineral, and the Ba concentration was the highest, because barite could easily form from the abundant SO_4_^2–^ ions in the hot spring water and, once formed, was a stable/intact mineral. Secular changes in the bulk geochemical compositions of the adsorption sheets exhibited four behaviors: (1) an exponential increase (e.g., As, Au, and Pb); (2) a linear increase (e.g., Fe, In, and Sn); (3) a linear decrease (only P); and (4) an exponential increase till ~ 12 h followed by a gradual decrease (e.g., Sc and Th) (Fig. 5 and Supplementary Table S1). These different behaviors indicated that some elements were continuously adsorbed onto the blue-green algal sheets during the in situ adsorption experiment, whereas the concentrations of other elements reached an adsorption limit. The linear decrease in the P concentration is attributable to the destruction and dissolution of the original P-bearing filamentous structure of the blue-green algae (Supplementary Figs. S1 and S2). The Au concentrations on the blue-green algal sheets were the highest after the 7-month experiment (10.35, 12.18, 15.70, and 30.01 ppm; Fig. 5 and Supplementary Table S1). The adsorption sheet attached to the bottom of the PFA basket had an Au concentration of 15.70 ppm, and the maximum value of 30.01 ppm was recorded on one of the adsorption sheets attached to the side of the PFA basket (Supplementary Table S4). Although in the laboratory experiment using the 0.5–100 ppm HAuCl_4_ solution with a pH of − 0.7 to 4 adjusted by the HCl solution or aqua regia at room temperature (~ 25 ℃), the limit of Au adsorption was reached in only ~ 10 h because of the formation of many Au nano-particles^16–18^, in the in situ adsorption experiments in the test water tank, the Au concentration was still increasing after 7 months (Fig. 5 and Supplementary Table S1). In the laboratory experiment using the HAuCl_4_ solution, the high initial concentration of dissolved Au induced fast nucleation followed by the growth of Au particles until the dissolved Au was used up^16–18,37^. In contrast, in the hot spring water in the test water tank, the dissolved Au concentration was extremely low (below the detection limit); therefore, the precipitation of Au was controlled by the limited initial nucleation, and overgrowth then occurred preferentially on the previously formed nuclei (Figs. 3c–f and 4). The Au concentration is highly correlated (correlation coefficient *r* > 0.7) with elemental concentrations of Sr (*r* = 0.75), Cd (0.71), Sn (0.76), Ba (0.74), Pb (0.76), and Bi (0.72), followed by Ag (0.68). These elements are well known to be enriched in sulfide and sulfate minerals, and such enrichment is consistent with our microscopic observations of abundant barite with minor galena (Fig. 3a, b).

The following elements had concentrations higher than 0.1 wt% in the hot spring water precipitate (sinter deposit) in the test water tank: Al (0.20 wt%), Ti (0.18 wt%), Fe (0.16 wt%), As (1.21 wt%), and Pb (0.55 wt%). In addition, the concentrations of Sb (295 ppm) and Ba (688 ppm) were relatively high (Supplementary Table S1). On the blue-green algal sheets, the concentrations of Ba and As were the highest and second highest, respectively, whereas in the hot spring water precipitate, those of As and Pb were the highest and second highest, respectively, and the Ba concentration (688 ppm) was one order of magnitude lower than the maximum Ba concentration (0.78 wt%) on the blue-green algal adsorption sheet. Thus, the blue-green algae induced selective and preferential adsorption and precipitation of minerals from the hot spring water. In particular, the Au concentration in the hot spring water precipitate was only 0.017 ppm, which is ~ 1/1700 of the maximum Au concentration on the blue-green algal sheets (~ 30 ppm) after 7 months. This result indicates that most of the Au in the hot spring water in the test water tank that was not adsorbed onto the blue-green algal sheet did not precipitate as Au-rich grains but was released downstream.

**Figure 6:**
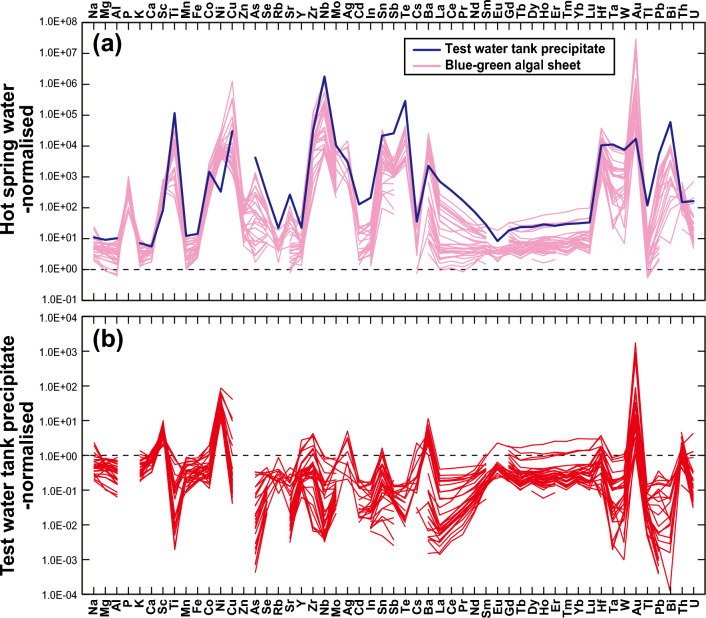
Bulk geochemistry enrichment results. Enrichment was calculated by normalizing the concentration on the blue-green algal sheets by the concentration in (**a**) hot spring water or that in (**b**) the sinter deposit in the test water tank. The average concentration of the six hot spring water samples (Supplementary Table S4) was used as the normalizing value. For Au only, the concentration in sample THS-UF3 was removed as an outlier when the average concentration was calculated. For concentrations of elements in the hot spring water below the detection limit, we used the detection limit to calculate the average concentrations. The enrichment factors of these elements may therefore be underestimates.

To understand the preferential adsorption onto the blue-green algal sheet, bulk geochemistry enrichment factors were calculated by normalizing concentrations to those in (1) the hot spring water or (2) the hot spring water precipitate (sinter deposit) in the test water tank (Figs. 6a, b). If element concentrations in the hot spring water were below the detection limit (< ** μg/L or ng/L; Supplementary Table S4), we used the detection limits to calculate the average compositions of the hot spring water for the purposes of normalisation (note that the Au value of sample THS-UF3 was an outlier and was removed from the calculations). Thus, the enrichment factors of those elements represent minimum values (Fig. 6a). The P and Zn concentrations in the hot spring water precipitate determined by ICP-MS were also below the detection limit; therefore, these data were also removed from the enrichment factor calculations (Fig. 6b). The enrichment factors calculated by normalisation to the hot spring water exceeded 10^5^ for five elements: Au was enriched the most relative to the hot spring water, by 3.0 × 10^7^; Ti was enriched by 1.0 × 10^5^, Cu by 1.3 × 10^6^, Zr by 1.3 × 10^5^, and Nb by 1.6 × 10^6^ (Fig. 6a). Other enriched elements included Ni (by 3.0 × 10^4^), Ag, (1.5 × 10^4^), Sn (3.5 × 10^4^), Te (4.6 × 10^4^), Ba (2.6 × 10^4^), Hf (3.9 × 10^4^), and Bi (1.1 × 10^4^). Among these elements, Zr, Nb, and Hf are compatible with refractory minerals such as zircon, whereas Cu, Au, Ni, Ag, Sn, Te, Ba, and Bi tend to be enriched in sulfide and sulfate minerals. Although 12 elements were enriched on the adsorption sheets more than 10^4^-fold relative to the hot spring water, only a few elements were more enriched on the adsorption sheets than in the hot spring water precipitate (Fig. 6a). Calculation of enrichment by normalisation to the hot spring water precipitate allowed clear visualisation of the elements that were released downstream or well adsorbed onto the blue-green algal sheet (Fig. 6b). Only four elements were enriched more than ten-fold relative to the precipitate: Ni (8.5 × 10^1^), Cu (4.0 × 10^1^), Ba (1.1 × 10^1^), and Au (1.7 × 10^3^). Among these four elements, Au was more enriched than the other elements by two orders of magnitude; this result demonstrates that the blue-green algal sheet showed strong preferential and selective adsorption ability for Au even from an Au-poor liquid such as hot spring water with an Au concentration below the detection limit. It should be noted that, when normalised to the hot spring water precipitate, As and Pb are not enriched onto the blue-green algal sheet, with their enrichment factors of less than 1 (Fig. 6b), indicating that these toxic elements were not preferentially and selectively adsorbed on the blue-green algal sheet.

The exponential weight loss of the absorption sheets during the experiment (Fig. 2) indicates that their durability needs to be improved for a more stable Au recovery method. Moreover, the adsorption behavior of the blue-green algae in an Au-poor solution with various combinations of dominant elements (components) should be investigated through more laboratory experiments, as well as the effective method of Au extraction from the blue-green algal sheet such as acid leaching and/or burning^18^. Nevertheless, our results demonstrate that our Au recovery method using an adsorption sheet made from blue-green algae has high potential for the future recovery of Au even from Au-poor solutions such as hot spring water, sewage water, mine wastewater, and seafloor hydrothermal fluid with a low impact on the environment.

## Conclusions

After a 7-month in situ adsorption experiment in acidic hot spring water, the Au concentration adsorbed onto the blue-green algal sheets was as high as ~ 30 ppm. The minimum enrichment of Au relative to its concentration in the hot spring water in the test water tank was estimated to be ~ 3 × 10^7^ fold; this value is one or two orders of magnitude higher than the enrichment value of other elements in the hot spring water. Furthermore, relative to the hot spring water precipitate, Au was enriched the most, by 1.7 × 10^3^, followed by Ni, Cu, and Ba. Thus, the blue-green algal sheets maintained their high preferential and selective adsorption ability for Au even from an Au-poor solution (Au below the detection limit) and in the presence of other major elements (Na, Mg, Al, K, Ca, and Fe > 20 ppm). Therefore, the Au recovery method using blue-green algal adsorption sheets has a high potential to recover Au from Au-poor resources.

## Methods

### Sample preparation for Au adsorption experiment

Blue-green algae collected from an alkaline hot spring in northeastern Japan were incubated in the laboratory in alkaline water (pH = 9–10) with liquid fertiliser at room temperature (~ 25 ℃)^17,18^. After incubation, the incubated blue-green algae were washed with tap water for 10 min several times to remove any remaining liquid fertiliser. Then, the algae were washed with a 2-M HCl solution for 10 min to remove unwanted components and enhance the Au adsorption ability and with tap water for 10 min several times to wash out the HCl solution and dried at 60 ℃ in an oven. The 7-month in situ adsorption experiment was performed from 15 October 2021 to 1 June 2022 because the Tamagawa Hot Spring is inaccessible during the winter because of heavy snow. The 2-month and 3-month experiments were begun on 9 August 2022, and the other, shorter term experiments were begun on 30 September or 1 October 2022. It should be noted that incubation batches of blue-green algae used in the in situ adsorption experiments and the starting and ending times of the experiments were not necessarily the same.

### Microscopic observations by FE-SEM

Scanning electron microscopy (SEM) imaging and energy-dispersive X-ray spectroscopy (EDS) analyses were performed in a Helios G4 UX system (Thermo Fisher Scientific Inc.) equipped with an EDS detector (Octane Elite Super (C5), Ametek) at JAMSTEC^30^. A 3-mm-diameter disc of a blue-green algal sheet was punched out and pasted onto an aluminum stub using double-sided carbon tape. Secondary electron (SE) imaging of the morphology of the adsorption sheets was performed at 1 kV, and SE and backscattered electron (BSE) imaging and EDS analyses of the constituent minerals were performed at an acceleration voltage of 10 or 20 kV without any conductive coatings. EDS elemental maps were obtained from L lines for Ba and Au, and from K lines for other elements; X-ray counts per second (CPS) maps were also obtained (Fig. 4 and Supplementary Fig. S3). For the SEM image shown in Supplementary Fig. S2b, the recovered sample was further rinsed with Milli-Q deionised water, dried under vacuum, embedded in epoxy resin, sectioned to a thickness of 1 μm using an ultramicrotome (UC7, Leica), and collected onto a copper grid. SEM–EDS imaging was performed at an acceleration voltage of 20 kV. The cross-correlation between the EDS maps of two elements A and B (I_XC_(A, B)) was calculated as follows:

$$I_{{{\text{XC}}}} \left( {{\text{A}},{\text{ B}}} \right)_{{{\text{ij}}}} { } = { }\left[ {I_{{\text{A}}} } \right]_{{{\text{ij}}}} { }\cdot{ }\left[ {I_{{\text{B}}} } \right]_{{{\text{ij}}}} /{{ \Sigma }}_{{{\text{ij}}}} { }\left( {\left[ {I_{{\text{A}}} } \right]_{{{\text{ij}}}} { }\cdot{ }\left[ {I_{{\text{B}}} } \right]_{{{\text{ij}}}} } \right)$$

where [I_X_]_ij_ denotes the intensity of element X at pixel (i, j)^38^.

### Major and trace element analyses by IC, ICP-AES, and (HR-)ICP-MS

Natural hot spring water at Obuki was collected on 14 October 2021 by the research group of Tohoku University. We brought back a hot spring water sample in a glass bottle to the laboratory without filtering or acid addition, because the pH of the hot spring water was low enough at 1.2. Hot spring water was collected from the test water tank on 30 September 2022 in two PFA bottles, one with filtering through an EMD Millipore membrane syringe filter (0.45 μm pore size, 25 mm diameter) and one without filtering (samples THS-F and THS-UF, respectively; Supplementary Tables S3 and S4). Each of the two hot spring water samples were then split into three subsamples for analysis (THS-F1–F3 and THS-UF1–UF3).

The mass fractions of the major constituents in the natural hot spring water collected at Obuki (Supplementary Table S2) were measured with an ion chromatography (IC) system (Dionex ICS-1600 or ICS-2100; Thermo Fisher Scientific Inc.) and an ICP-AES system (SPECTRO ARCOS; SPECTRO Analytical Instruments Inc.) at JAMSTEC after appropriate sample filtering and dilution with Milli-Q deionised water, following the method of Miyazaki et al.^33^. These results were calibrated by using diluted reference solutions made with the ICP Multi-element Standard IV Certipur® (Merck), the Anion Mixture Standard Solution 1, the Cation Mixture Standard Solution III, the Chloride Ion Standard Solution (Cl^–^ 1000) and the Sodium Standard Solution (Na 1000) (FUJIFILM Wako Chemicals).

Major and trace element analyses of the blue-green algal sheets and the hot spring water in the test water tank (Supplementary Tables S1 and S3) were performed by ICP-MS with an Agilent 7500ce system installed at JAMSTEC. Powdered samples weighing ~ 50 mg (1 mL of hot spring water) were dissolved by the HNO_3_-HClO_4_-HF digestion method in Teflon PFA screw-cap beakers and then heated overnight on a hot plate at 110 ℃. The digested samples were progressively evaporated at 110 ℃ for more than 12 h, at 130 ℃ for 3 h, and at 160 ℃ until dryness. The residue was dissolved in 5 mL of Milli-Q deionised water combined with 4 mL HNO_3_ and 1 mL HCl, and then further diluted to 1:100 or 1:20 by mass (total dilution factor ~ 20,000 or ~ 200) before introduction into the ICP-MS system. Details of these analytical procedures, including instrumental drift and mass interference correction methods, are reported in Takaya et al.^31^ and Nozaki et al.^32,34^.

The hydrogeochemistry of the hot spring water in the test water tank (Supplementary Table S4) was also analysed by ICP-MS or HR-ICP-MS at Activation Laboratories Ltd., Canada, using the following packages: Code 6 (Total Recoverable Natural Waters with low TDS [total dissolved solids] [< 0.05%]), Code 6 Overrange (Overrange elements in Code 6 MB [marine water, brines, or other aqueous solution with TDS > 0.05% reanalysed by ICP-AES or ICP-MS if required]), Code 6 HR-ICP-MS (Water analysis by High Resolution ICP-MS), Code 6 Au HR-ICP-MS (Au by High Resolution ICP-MS), and Code 6 Boron (Boron add-on by ICP-MS).

### Supplementary Information

Supplementary Figures.

Supplementary Table S1.

Supplementary Table S2.

Supplementary Table S3.

Supplementary Table S4.

## Data Availability

All data generated or analysed during this study are included in this published article and supplementary information files.
