# Supplementary material for: In situ gold adsorption experiment at an acidic hot spring using a blue-green algal sheet
Source: Sci Rep. 2024 Mar 8;14:5739. doi: 10.1038/s41598-024-56263-3 (PMC10923829; doi:10.1038/s41598-024-56263-3)
Supplement: Supplementary file 1 — Supplementary Figures. [file 41598_2024_56263_MOESM1_ESM.pdf]

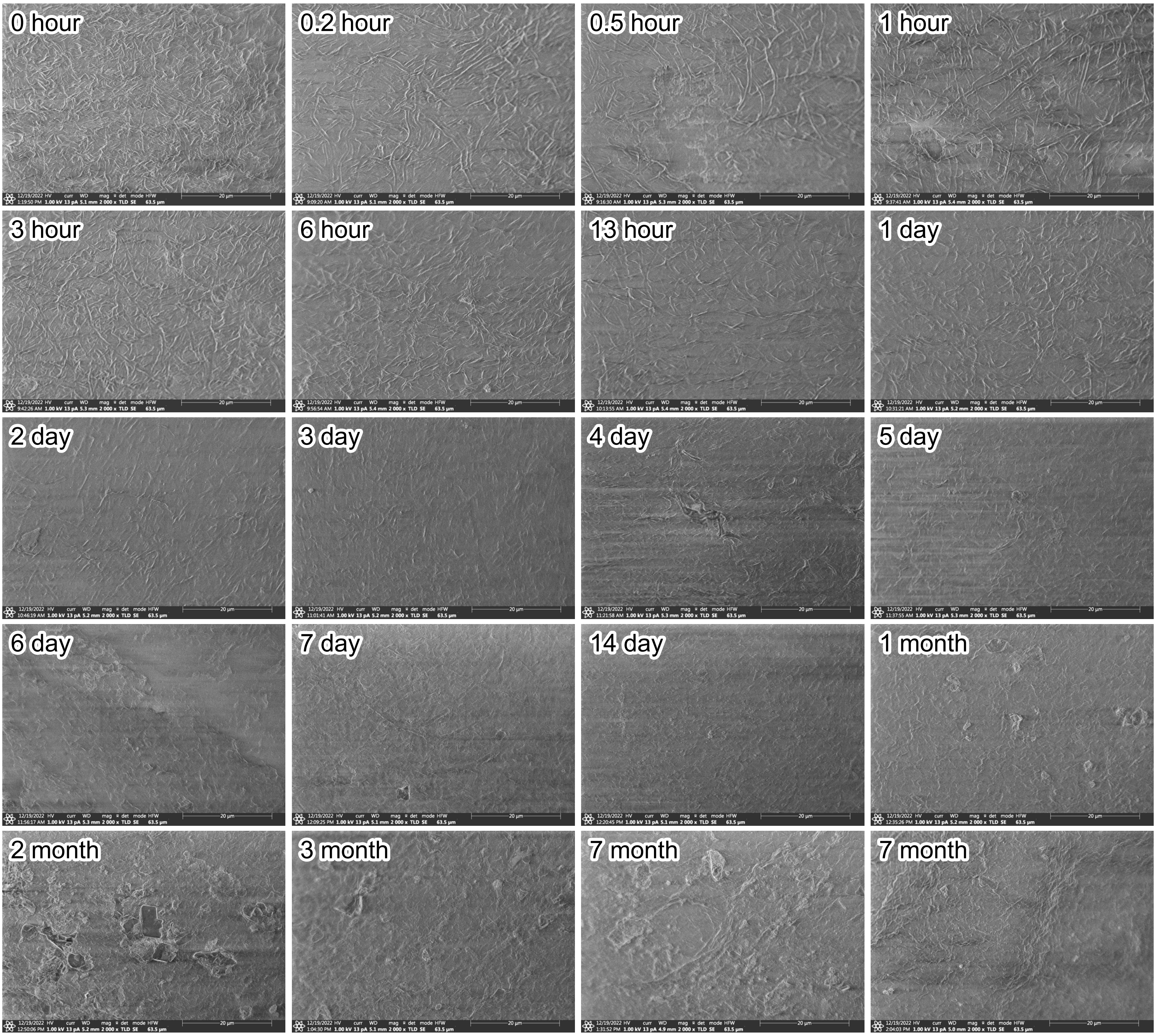

Supplementary Figure S1 SEM images of blue-green algal sheets after various reaction times. The blue-green algal sheets preserved the original filamentous algal structure until day 7. Subsequently, the filamentous structure became vague and unclear, and the occurrence of precipitated minerals (dominated by barite) increased on the sheets.

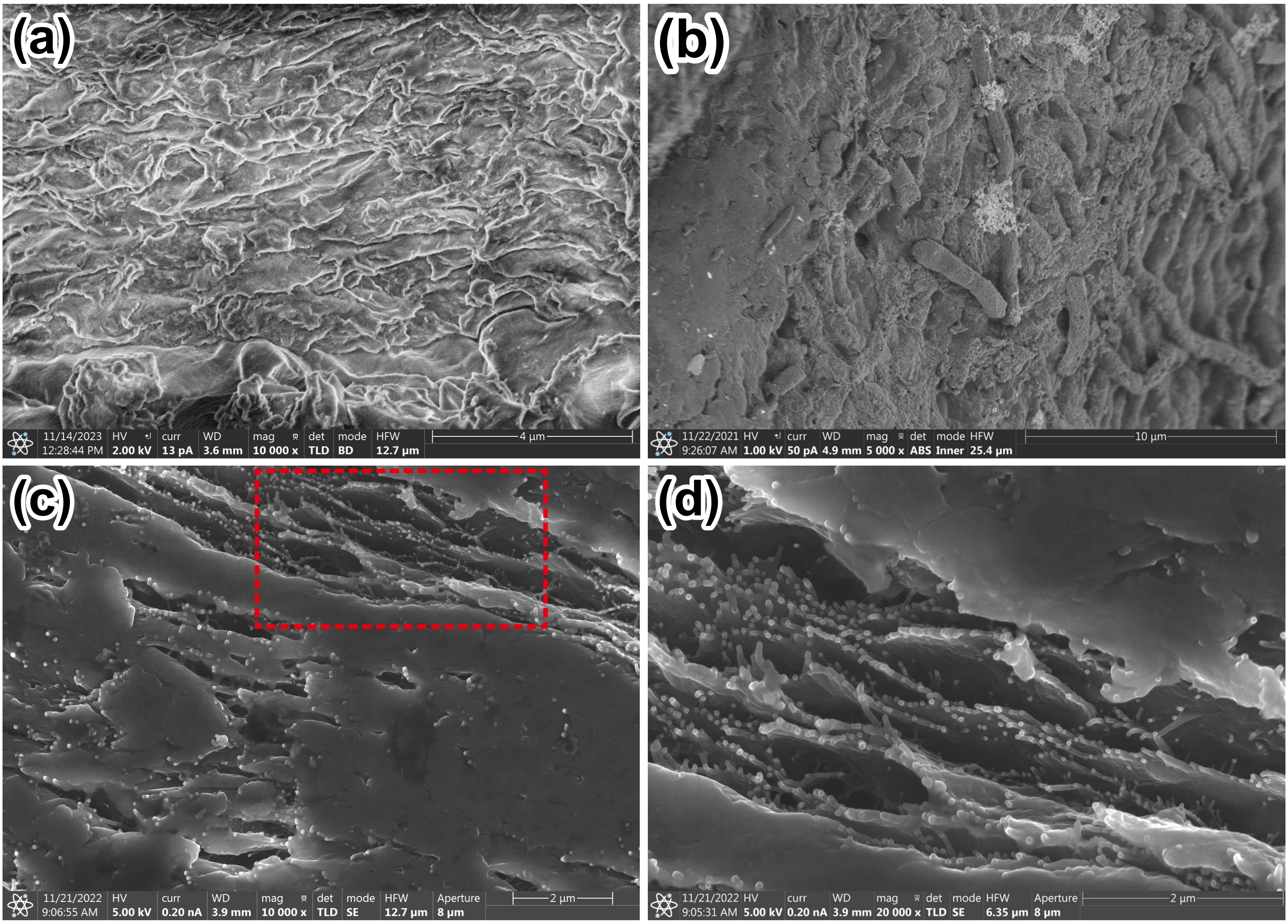

Supplementary Figure S2 SEM images of blue-green algal sheets: (a) Cross-section of the as-prepared sheet. (b) After a 20-h Pd adsorption experiment conducted in the laboratory. In this experiment, ~0.2 g of blue-green algae and ~100 ppm of Na_2PdCl_4 solution were rotated at 300 rpm in a glass beaker at room temperature. This SEM image was taken after the short term Pd (not Au) adsorption experiment, but is shown as the representative filamentous structure of blue-green algae before the long term metal adsorption experiment. (c) Surface structures of a blue-green algal sheet after the 7-month *in situ* Au adsorption experiment in the test water tank at Tamagawa Hot Spring. (d) Enlarged image of the area in the dotted red rectangle in (c).

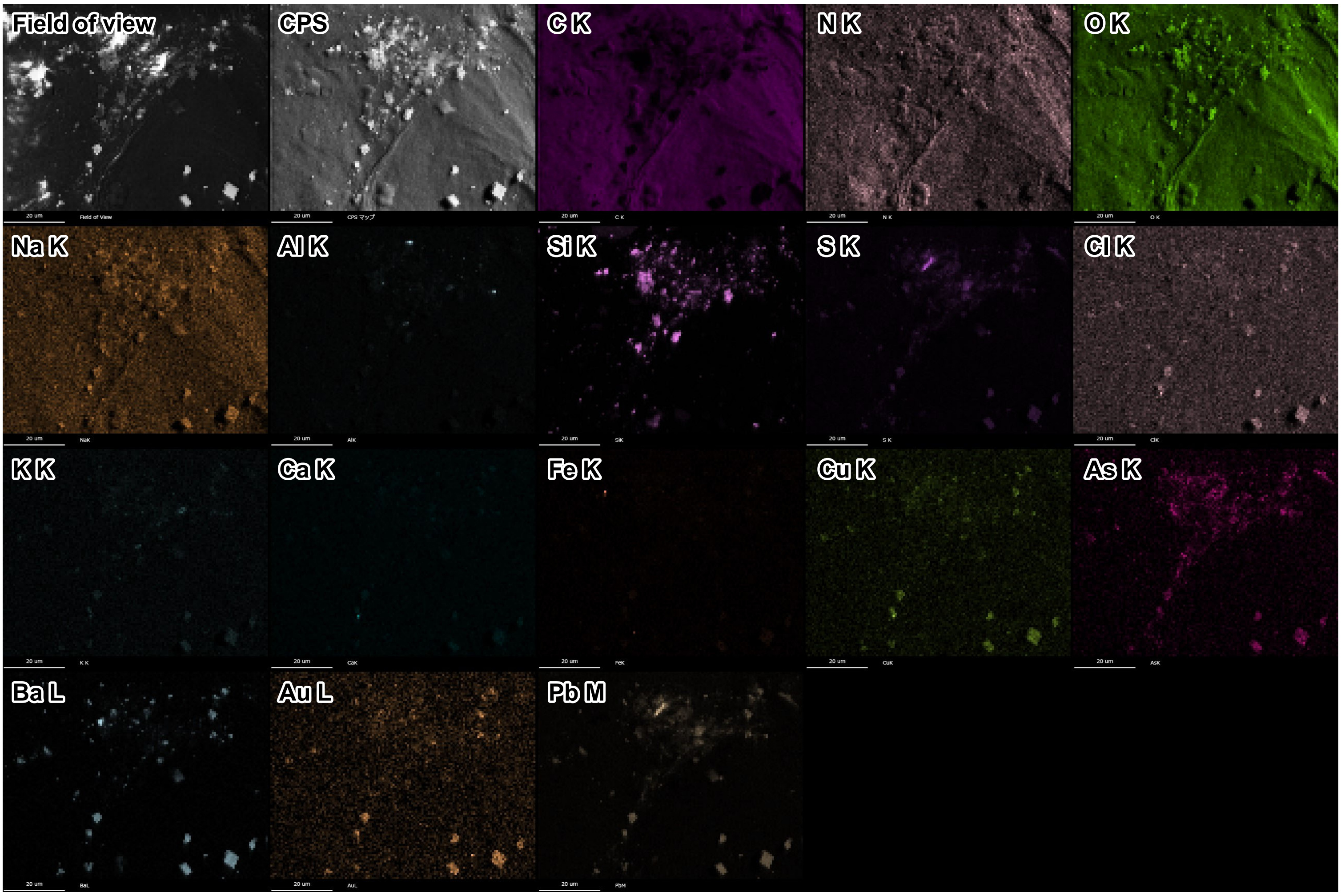

Supplementary Figure S3 SEM-EDS elemental mappings. EDS elemental maps after the 7-month *in situ* Au adsorption experiment were obtained from the L lines for Ba and Au, the M line for Pb, and the K lines for other elements. An X-ray counts per second (CPS) map was also obtained. White scale bars indicate 20 μm.
